# The Functional Role of MnSOD as a Biomarker of Human Diseases and Therapeutic Potential of a New Isoform of a Human Recombinant MnSOD

**DOI:** 10.1155/2014/476789

**Published:** 2014-01-06

**Authors:** Antonella Borrelli, Antonietta Schiattarella, Patrizia Bonelli, Franca Maria Tuccillo, Franco Maria Buonaguro, Aldo Mancini

**Affiliations:** Molecular Biology and Viral Oncology Unit, Department of Experimental Oncology, Istituto Nazionale per lo Studio e la Cura dei Tumori “Fondazione G. Pascale”—IRCCS, Naples, Italy

## Abstract

Reactive oxygen species (ROS) are generated as a consequence of metabolic reactions in the mitochondria of eukaryotic cells. This work describes the role of the manganese superoxide dismutase (MnSOD) as a biomarker of different human diseases and proposes a new therapeutic application for the prevention of cancer and its treatment. The paper also describes how a new form of human MnSOD was discovered, its initial application, and its clinical potentials. The MnSOD isolated from a human liposarcoma cell line (LSA) was able to kill cancer cells expressing estrogen receptors, but it did not have cytotoxic effects on normal cells. Together with its oncotoxic activity, the recombinant MnSOD (rMnSOD) exerts a radioprotective effect on normal cells irradiated with X-rays. The rMnSOD is characterized by the presence of a leader peptide, which allows the protein to enter cells: this unique property can be used in the radiodiagnosis of cancer or chemotherapy, conjugating radioactive substances or chemotherapic drugs to the leader peptide of the MnSOD. Compared to traditional chemotherapic agents, the drugs conjugated with the leader peptide of MnSOD can selectively reach and enter cancer cells, thus reducing the side effects of traditional treatments.

## 1. Introduction

Reactive oxygen species (ROS) are produced during normal cellular function [1]. The effect of ROS on cell fate depends on the level at which ROS are present [2]. ROS are extremely reactive and unstable. This chemical reactivity induces lipid peroxidation and protein oxidation and degradation [3]. There are three main types of ROS: superoxide anion radical (O_2_^∙−^), constitutively present in cells and is due to the respiratory chain in mitochondria; hydrogen peroxide (H_2_O_2_), derived from the dismutation of (O_2_^∙−^) or directly from the action of oxidase enzymes; and hydroxyl radical (^∙^OH), a highly reactive species that can modify purine and pyrimidine bases and cause strand breaks that result in DNA damage [4]. ROS can induce DNA sequence changes in the form of mutations, deletions, gene amplification, and rearrangements [5]. The result of these structural DNA modifications may be the activation of oncogenes and the inactivation of suppressor genes [6]. While healthy cells require low-level concentrations of ROS in order to signal transduction before their dismutation, cancerous cells need high levels of ROS to keep their rate of proliferation high. While normal cells reduce their low levels of ROS through aerobic respiration in the mitochondria, cancer cells deal with their large amounts of ROS using alternative pathways such as the glycolytic pathway into the pentose phosphate pathway (PPP) and/or the generation of lactate. ROS levels can be effectively used to monitor the damage that cells can tolerate [7]. Unbalanced levels of ROS and endogenous antioxidants are related to many disorders, including central nervous system pathologies [8–13] (e.g., Parkinson's disease [12], Alzheimer's disease [10], cardiovascular conditions [10, 11], pulmonary diseases [14, 15], diabetes [16, 17], ocular diseases [18, 19], aging [20–22], cancer [23–25], and radiation damage [26]). Reactive Oxygen Species, control the inflammatory and immune responses by acting on the cell's transcriptional activity [27–36]. Oxidative stress is not considered a disease, but it is correlated to many of them [37]. There are more than 42,000 publications on superoxide radicals and the dismutation enzymes.

## 2. Superoxide Dismutases (SODs) and Their Activities

Antioxidant enzymes are compartmentalized and the activity is controlled by genetic regulation. These enzymes include superoxide dismutase (SOD), glutathione peroxidase, catalase, and peroxiredoxin. Main biochemical reactions produce and scavenge reactive oxygen species (ROS). Superoxide dismutase reacts with the superoxide anion to form hydrogen peroxide and molecular oxygen. Catalase converts hydrogen peroxide to water and molecular oxygen. Catalase also reacts with hydrogen donors (methanol, ethanol, formic acid, phenol) using 1 mol of peroxide for peroxidase activity. Glutathione peroxidase catalyses reduction of a variety of hydroperoxides using reduced glutathione. Fenton reaction produces hydroxyl radicals.

### 2.1. Types of SODs

The SODs are a family of enzymes that very efficiently catalyze the dismutation of the superoxide radical anion (O_2_^∙^^−^): O_2_^∙^^−^ + O_2_^∙^^−^ + 2H^+^ →H_2_O_2_+O_2_. Superoxide was discovered in the 1930s by Pauling [38]. A few years later, Mann and Keilin [39] purified the protein from bovine blood and liver as a copper-binding protein of unknown function. The protein was called “erythrocuprein” or “hepatocuprein” or later “cytocuprein.” The purification was based solely on copper content. Huber et al. [40] isolated the same protein from bovine liver in the 1960s based on its anti-inflammatory activity in animal models. They called the protein orgotein. Knowles et al. [41] in 1969 showed that the enzyme xanthine oxidase could indeed produce superoxide. The discovery of the enzymatic activity of the SODs was reported in 1968–1969 by McCord and Fridovich [42, 43] who showed that the copper protein of Mann and Keilin could catalytically eliminate the Pauling free radical. SODs are present everywhere in living aerobic organisms. SODs can come with four different types of metal centers: Cu/Zn (aka SOD1), Ni, Mn (aka SOD2), and FeSODs. SODs can be intracellular or extracellular. The extracellular SOD (aka SOD3) comes with Cu/Zn metal center. The most important SODs in man are SOD1, SOD2, and SOD3. Cu/Zn SOD is generally homodimeric and is present in diverse locations in different organisms. It is found in the periplasm of bacteria (sod-C), cytoplasm and chloroplasts of plants, and several compartments such as nucleus, lysosomes, peroxisomes, cytosol, and extracellular milieu in animals [44]. Copper, zinc superoxide dismutase, are a class of enzyme preserved throughout evolution, which usually have two identical subunits of about 32 kDa, each containing a metal cluster, the active site, constituted by a copper and a zinc atom bridged by a common histidine ligand [45]. SOD1 also acts, but less efficiently, as a nonspecific peroxidase [46]. The induction mechanisms of SOD1's gene have been discussed [47]. Cells hardly uptake SOD1. SOD1 appears to be a very important enzyme for the prevention of aging and mutation by oxidative stresses and hazardous effects from environmental factors [48]. Extracellular superoxide dismutase (EC-SOD) is a secretory, tetrameric, copper- and zinc-containing glycoprotein found in the interstitial spaces of tissues and equally in extracellular fluids. Antioxidative activity in plasma, lymph, and synovial fluid is due to EC-SOD [49]. It is the major antioxidant in the blood vessel wall interstitium [50]. It is the only known extracellular enzyme designed to scavenge (O_2_^∙^^−^) [51]. EC-SOD's regulation is coordinated by cytokines and is not induced by a cell reaction to ROS [52]. EC-SOD has primary control over the inactivation of nitric oxide and plays an important role in neurobehavioral function in controlling oxidative stress and intercellular signaling [53, 54].

### 2.2. MnSOD (SOD2) as Novel Marker of Cancer

Manganese superoxide dismutase (MnSOD) is known to have important functions in a broad range of stress-induced diseases. MnSOD is the only enzyme that is essential for the survival of life in an aerobic environment under physiological conditions [55, 56]. MnSOD is essential for life, whereas Cu, ZnSOD is not [57]. This critical function may be due to the strategic location of MnSOD in the mitochondria. MnSOD is a nuclear-encoded enzyme that is very highly regulated. The expression of MnSOD can be regulated at multiple levels from transcription and translation to posttranslational modifications. Various extracellular and intracellular factors induce MnSOD expression and modulate its activity [58]. MnSOD has attracted the attention of many researchers, since the expression of the mitochondrial form has been found altered in cancer, as well as in other diseases. The MnSOD genes from human, bovine, rat and mouse share more than 90% homology in the coding sequence [59]. The SOD2 gene is located on chromosome 6q25 [60]. It is frequently deleted in several types of human tumors. The association of the 6q deletion with the diminution of MnSOD suggests that it might be a new type of tumor suppressor [61], although it has been suggested that the deficiency of enzyme activity could be due more to a defect in MnSOD's gene expression than to its deletion [62]. Moreover, a study performed in 1991 with human colon cancer cells and SV40-transformed human lung fibroblasts showed that the reduced level of MnSOD is due to neither a defect in the primary structure of MnSOD protein nor a decrease in MnSOD mRNA stability [63]. It was also found that a decrease in MnSOD expression and enzymatic activity depends on the preservation of the chromosome 6q25's long arm [58]. MnSOD is elevated in cancer cells compared to their normal counterparts, including gastric and esophageal [64], lung [65], and colorectal [66] cancer cells. When there is a higher level of MnSOD in the cancer cells, the aggressiveness of cancer and its metastatic potential [67] are increased, and the prognosis is poor [68]. While in some types of tumor the levels of this protein have been shown to inversely correlate with pancreatic cancer cell growth [69], other studies show that MnSOD is reduced in many types of cancer [70], including breast cancer [71, 72], pancreatic cancer [73], and ovarian cancer [74]. The onset of cancer usually involves alterations in the control of cell growth and proliferation, DNA damage, and reactive oxygen species (ROS) production. MnSOD, when overexpressed, inhibits many of the typical properties of cancer (increased growth rate, invasiveness, and anchorage independent cell growth) [75–78]. MnSOD plays a role as a tumor suppressive protein, which inhibits cell proliferation and intensifies apoptosis [79]. It influences the activity of some transcription factors, such as activator protein 1 (AP-1), nuclear factor-kappa B (NF-*κ*B), and p53 [80, 81]. MnSOD also protects the normal tissues from chromosomal instability, due to various injuries, causing cancer [82]. Moreover, this enzyme modulates the ROS concentration in cancer cells [83, 84]. The tumor suppressive effects of MnSOD have been studied in correlation to altered ROS levels [85–87]. When overexpressed, MnSOD weakens cancer cells, which become more vulnerable to ROS-generating agents and die both in vitro and in vivo [88]. Overexpression of an active site mutant of MnSOD delays growth of the human embryonic kidney cells HEK293 [89]. Ridnour et al. show a decrease of tumorigenicity of rat embryo fibroblast cells through MnSOD overexpression [85]. Such decreased tumorigenicity correlates MnSOD's activity to hydrogen peroxide. The same results were found in PC-3 human prostate cancer cells, in which higher levels of hydrogen peroxide were correlated with MnSOD's overexpression and decreased cell growth [76]. In a study by Jang et al. [90], the effects of MnSOD overexpression on age-related biomarkers were investigated. When overexpressed, MnSOD protects mice from paraquat-induced oxidative stress and causes an increase in aconitase activity. A decrease of MnSOD was also correlated to age-related pathologies.

### 2.3. SOD as a Drug

Despite the findings on the role of superoxides, clinical use of therapies based on superoxide-scavenging mechanisms, whether by use of SOD as a drug, or by SOD mimetics, or by stoichiometric scavengers of the radical, is still far from being widespread for a number of reasons. An example is diabetes; although it is evidently associated with high levels of oxidative stress [91], few diabetic patients are advised to supplement their diet with any form of antioxidants; moreover, biochemical markers of oxidative stress are not monitored in such patients. Only few major pharmaceutical companies are reported to carry out R&D projects addressing oxidative stress [37]. The use of SODs as drugs met several technical difficulties related to their chemical nature. SODs, like any other enzymes used as drugs, have a charge density and are characterized by rapid renal clearance that negatively affects pharmacodynamics and pharmacokinetics. Recently, a genetically engineered version of human SOD2 has been described that may overcome some of these problems [92]. Some researchers have tried to solve the problem, using liposomes as carriers of SOD with some success [93–95]. Another reason why SOD-based antioxidant therapy has not yet made a greater impact on clinical medicine may be that its effects are represented by a bell-shaped curve. SODs are very effective up to a point, beyond which they lose their protective activity and may even exacerbate the condition. A frequent consequence of bladder irradiation is fibrosis, a physiological response that often follows inflammation in other tissues. Clinical trials of SOD have been carried out to treat bladder inflammation resulting from irradiation leading to fibrosis. SOD was used very early in [96–98]. Promising results have emerged from a study on kidney transplant patients [99]. SOD reduced acute rejection episodes (from 33.3% in controls to 18.5% in SOD-treated), as well as early irreversible acute rejection (from 12.5% in controls to 3.7%). Moreover 74% of SOD-treated patients survived (with a projected half-life of 15 years) compared with 52% in controls (with an extrapolated half-life of 5 years). SOD appears to play a beneficial role in immunosuppression and in graft rejection. The immune system is able to respond to “danger signals” resulting from oxidative stress [100]. Superoxide radical is being increasingly viewed as a signaling molecule, especially with regard to cell division and proliferation [101, 102]. SOD and oxidative mechanisms are involved in transformation, metastasis, and angiogenesis, causing lipid peroxidation, protein oxidation, and DNA damage [103–105]. The implications for ROS regulation are highly significant for cancer and other disease therapies because commonly used radio and chemotherapeutic drugs influence tumor outcome through ROS modulation. Some researchers are now using adenovirus containing SOD and CAT in the treatment of various cancers both in vitro and in vivo [106, 107]. Overexpression of MnSOD inhibits ras-induced transformation, modulated by intracellular ROS level [108]. A recombinant adenoviral vector expressing MnSOD has been used, showing the antitumor effect by itself, but this effect is much more evident when using anticancer drug 1,3-bis(2-chloroethyl)-1-nitrosourea (BCNU) [109]. A phase I study has been done on human cancer protection with an intravesical injection of MnSOD plasmid liposomes [110]. To achieve local tumor control to minimize radiation toxicity, a radioprotective gene therapy was tested with MnSOD plasmid liposome [111]. The same MnSOD plasmid liposome was used in human intratracheal gene therapy in order to decrease pulmonary radiation resistance [112]. It is noteworthy that the protective effect of MnSOD has already been widely documented in the literature, but most studies use bovine protein. While it has been evident that SOD was able to protect organisms from ischemic conditions, in none of these was the result less than satisfactory because of its bovine origin. Also, it was not considered injectable, as biological barriers lead to its rapid inactivation. The same thing also occurred when the constructs of human MnSOD genes bound to viral probes were administered. The present line of work describes the obtainment, the initial application, and the many possibilities of a form of human MnSOD which is easily injectable. Even the isolated leader peptide alone may have many relevant applications in the biomedical field, as both a diagnostic and therapeutic molecular tool.

## 3. A Novel Recombinant Manganese Superoxide Dismutase (rMnSOD)

**Figure 1:**
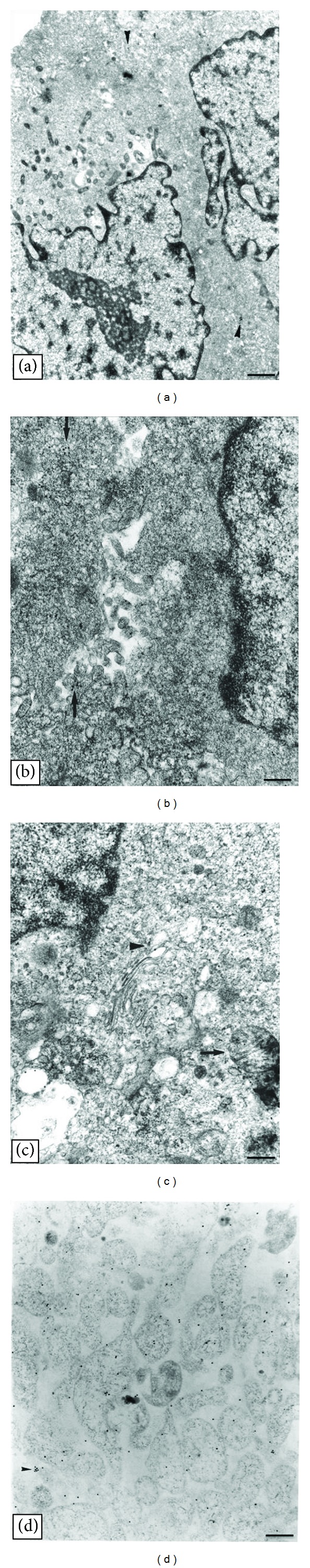
Transmission electron microscopy of LSA cells. Immunogold method: 15 NM colloidal gold antibody, (a) Low magnification of three LSA cells showing the contact among them and the secretion vesicles containing LSA-MnSOD (arrow head) Scale bar (in D): 150 nm. (b) Note the clustered colloidal gold particles in the secretion vesicles and in RER of LSA cell (arrows). Scale bar (in D): 450 nm. (c) Detail of LSA cell showing LSA-MnSOD (demonstrated by the colloidal gold particles) in Golgi vesicles (arrow head) and in mitochondria too (arrow). Scale bar (in D): 450 nm. (d) TEM section of LSA cell without osmium tetroxide to preserve immunogenicity. Immunoreaction for LSA-MnSOD revealed by 15 nm colloidal gold. Note the abundance of colloidal gold particles in the secretory vesicles. Scale bar: 45 nm.

Modified isoforms of SOD are present in various tumors and take part in the autocrine mechanisms of cell-growth inhibition. On the other hand, it is well known that the interaction between tumors and neighboring tissues plays a key role in the process of oncogenesis. The manganese superoxide dismutase from liposarcoma (LSA-MnSOD) is a tumor protein isolated and sequenced, for the first time, in human liposarcoma (LSA) cells [113]. The LSA cells secrete factors in the culture medium, which can exert a cytotoxic activity on the human mammary tumor cell line MCF-7. In 1991, Mancini et al. [114] successfully cultured and cloned an adipocyte cell line from a human liposarcoma (LSA). In 1999, Mancini et al. [115] managed to have a continuously growing cultured cell line in vitro, from ascites fluid belonging to a patient with a poorly differentiated ovarian adenocarcinoma. The growth of these cells was inhibited by a new regulatory activity, present in the liposarcoma cell line, which arrested their cell cycle in the G1 phase and induced them to apoptosis. The reciprocal action between breast carcinoma cells and liposarcoma cells was studied using an in vitro model. The liposarcoma-derived cell line (LSA) secreted a factor which was able to kill some tumor-derived mammary epithelial cells but not the corresponding MCF-10 normal mammary epithelial cells [116]. This factor was the LSA-type-MnSOD. Immunocytochemical detection of MnSOD in LSA cell, by using a colloidal gold anti-MnSOD antibody, showed that MnSOD is located in RER, mitochondria, and secretion vesicles of LSA cells (Figure 1).

### 3.1. The Oncotoxic Effect of rMnSOD

The identification of the oncotoxic component of the secrete was obtained by a sequence of chromatographic purification steps of the LSA cell culture medium supernatant. For each chromatographic fraction, the cytotoxic activity on MCF-7 cells was tested and the protein content was analyzed by SDS-PAGE. The result was the isolation of a single protein band with a molecular weight of about 30 KDa, which showed increasing biological activity along with purification steps. The polypeptide was digested with the protease LysC and then sequenced by MALDI-MS/MS. The amino acid sequences were “GELLEAIK” and “GDVTAQIALQPALK,” which, in BIOMED, aligned with human MnSOD to 100% and 92.8%, respectively. The presence of minor components (14-3-3 proteins) was detected together with the MnSOD in the LSA cell culture medium. In order to assess whether the oncotoxic activity was due to MnSOD or to the minor components, a test of immunodepletion of the putative cytotoxic components was performed, using antisera against human MnSOD, 14-3-3 proteins, or MMP-1 as a negative control. The single immunodepleted components were tested on the MCF-7 cells, to evaluate their cytotoxic properties. This experiment demonstrated that the cytotoxic component contained in the LSA cell culture medium is just the MnSOD, which was able to kill cancer cells within 3 hours after application [113].

Mancini et al. then demonstrated that the unique difference between the Wild Type protein and its mutant variant, extracted from the LISA cells conditioned medium, was that the latter permanently retains the “Signal Peptide,” otherwise known as “Leader Peptide” (LP), a short (typically 5–30 amino acids long) and very hydrophobic amino acid sequence present at the N-terminus end of most newly synthesized secretory proteins.

### 3.2. The Oncotoxic Mechanisms of rMnSOD

Normally the secretory proteins are correctly directed to their destination already during their synthesis (which always starts at the N-terminus end of the molecule) by means of the LP, which is then promptly cleaved and thus never found in the amino acidic sequences of fully matured proteins and peptides. The abnormal persistence of the LP, evidently never cleaved, allows this mutant protein to freely move through the hydrophobic environment of cellular membranes, either internal or external. This unique feature, key for its therapeutic potential in many fields of biomedicine, is also retained by the recombinant mutant protein (rMnSOD), which was obtained by Dr. Mancini through genetic engineering techniques once the gene had been sequenced. The rMnSOD, unlike the Wild Type protein, when injected, can thus easily permeate virtually all cells and organs and exert there its enzymatic activity. The selective antitumor effect of the rMnSOD is possibly due to the low catalase activity present in most tumor cells (typically 10 to 50% lower than in normal cells). The hydrogen peroxide (H_2_O_2_) enzymatically produced inside the cell from free radicals and reactive Oxygen species by the rMnSOD may thus specifically damage tumor cells. In contrast, the high levels of catalase typically present in normal cells may promptly eliminate most of the SOD-produced H_2_O_2_ by readily transforming it in O_2_ and H_2_O, clearly not toxic. Normal cells may even benefit from the extra molecular oxygen generated by this reaction [117].

### 3.3. rMnSOD and Radiation Biology: The Radiosensitizer and Radioprotective Action

The ionizing radiation, because of its effect on the cytoplasmic water, produces huge amounts of free radicals, generating a strong oxidative stress. Borrelli et al. demonstrated that rMnSOD exerts a radioprotective role on normal cells and tissues exposed to lethal doses of ionizing radiation, while acting as a radiosensitizer for tumor cells [118].

### 3.4. The Function of Molecular Carrier of Leader Peptide

More recently, Dr. Mancini and collaborators investigated the possible applications of the effective and rapid internalization of the modified molecule in cells, in striking contrast with the Wild Type protein, due to the fact that the persisting LP of the rMnSOD acts as its molecular carrier. A synthetic preparation of this peptide, composed of 24 amino acids, was conjugated to the antitumor drug cisplatin. This construct, carrying a modest amount of cisplatin (11 micrograms), was either added to in vitro cultured cells or injected in spontaneous tumor-bearing animals. They thus demonstrated that the peptide penetrated in practically all tumor cells, releasing approximately a total of 80 nanograms of Platinum and causing their immediate death. In control cells and animals to which 11 micrograms of Cisplatin alone were given, the total platinum concentrations only reached 3 to 6 nanograms in similar tissue masses. They thus concluded that, by using the LP as a molecular carrier, significantly lower quantities of antitumor agents may be administered to tumor-bearing patients; this would result in a substantial increase of antitumor action and thus of the therapeutic margin. It should be emphasized that this method could transform generically antireplicative molecules (such as cisplatin or other cytostatics) into specific antitumor drugs [119, 120].

### 3.5. Antifibrotic and Ischemic Protective Effect of rMnSOD

Very recently, rMnSOD—being easily injectable in vivo and immediately available and active in virtually all tissues—has been used in several experimental animal models. In those, rMnSOD reduced by 90% the value of portal hypertension in cirrhotic rats and also significantly controlled the degree of liver fibrosis. By removing the free radicals present in high concentration in liver vessels, it allows the endothelial cells of these vessels to reuse nitric oxide (NO). In this way the endothelial cells, by reactivating their normal functions, resume their responses to vasodilatory and vasoconstrictor stimuli. This resulted in the disappearance of ascites and in considerable reduction of liver fibrosis [121]. Moreover, rMnSOD allowed a 80% rescue of kidney glomerular filtrates in rats which had received high doses of Cyclosporin-A. Given the well-known, potent ischemic effect of this drug, rMnSOD could be useful in the treatment or prevention of the kidney ischemic damages caused by prolonged immune suppressive therapies with Cyclosporin-A or by antitumor drugs [122]. The group has also demonstrated that rMnSOD may also protect the kidneys by X-ray contrast media [123].

## 4. Conclusions

New modalities of treatment for cancer are needed, because the majority of patients continue to die of metastasis despite an initial response to conventional chemotherapy [124, 125]. Anticancer therapies may be efficient in early stages of the disease, but advanced tumors are usually resistant to the same treatments [126]. Over one hundred thousand researchers worldwide are actively investigating the importance of signal transduction in cancer development [127]. Many groups are studying the possibilities of antioxidant therapy [128]. The therapeutic use of antioxidants may involve the use of naturally occurring antioxidants or completely synthetic molecules [129]. There is also evidence that some drugs already used clinically may exert part of or all their effects by antioxidant mechanisms [130]. Against this background, small molecules that mimic antioxidant enzymes [131–133] are becoming new tools for the treatment of many diseases [134, 135].

Poor permeability and selectivity of the cell membrane are an important obstacle to the development of peptide drugs and therapeutic proteins [136] because of the endothelium, which is the first physiological barrier between blood and tissues. Moreover, the endothelium can be easily injured by drugs [137]. To deliver full-length proteins into a large number of cells, researchers are designing new nanotech strategies [138] using nanoparticles for the delivery of antineoplastic drugs such as adenoviral vectors to improve gene transfer into tumors, lipid-based nanostructures, micelles from amphiphilic block copolyphosphates, antibody-drug conjugates; biodegradable polyester or polyethylenimine nanoparticles approved for human use, and graphene oxide-based noncovalent nanosupramolecular complexes [139–147].

The functional studies on rMnSOD show that it plays a role as a specific, targeted cytotoxic agent only on cancer cells. Its leader peptide is a good carrier of cytostatic drugs and allows them to enter cancer cells in higher amounts and induce apoptotic death, so that lower amounts of chemotherapic drugs can be used, thus reducing the side effects. Moreover the administration of rMnSOD results in a protective effect on normal cells. It is important to note that, despite a positive uptake of rMnSOD-Lp-CC by normal cells, the apoptotic reactions were undetectable, as was confirmed by LDH release and by Bax gene expression [119, 120].

These differences in uptake can be related to the fact that the low amount of cisplatin contained in rMnSOD-Lp-CC may be insufficient to reach the toxicity threshold, given that in normal cells there are normal levels of antioxidant enzymes (MnSOD and catalase), sufficient for the normal cells to neutralize the cytotoxic effect of cisplatin derived free radicals, reducing the secondary side effects of chemotherapic treatment. In contrast, the amount of cisplatin taken up by cancer cells will be very toxic for them given the modest quantity of protective enzymes, particularly catalase, they express. Antireplicative drugs have major limitations in cancer therapy; in fact they are not specific to cancer cells and exert their cytotoxic activity not only on tumor cells, but also on most of normal replicating cells. The leader peptide, instead, as a carrier of cytostatic drugs, allows the chemotherapic drugs with a generic antireplicative activity acquire a specific and selective antitumor activity, greatly improving patient survival.
